# Epidemiology of human papillomavirus-associated anogenital cancers in Granada: a three-decade population-based study

**DOI:** 10.3389/fpubh.2023.1205170

**Published:** 2023-09-14

**Authors:** Pablo Dabán-López, Nicolás Francisco Fernández-Martínez, Dafina Petrova, Miguel Rodríguez-Barranco, Jose Juan Jiménez-Moleón, Javier Gutierrez, María-José Sánchez

**Affiliations:** ^1^Servicio de Cirugía General y del Aparato Digestivo, Hospital Universitario San Cecilio, Granada, Spain; ^2^Instituto de Investigación Biosanitaria ibs.GRANADA, Granada, Spain; ^3^Escuela Andaluza de Salud Pública (EASP), Granada, Spain; ^4^CIBER of Epidemiology and Public Health (CIBERESP), Madrid, Spain; ^5^Department of Preventive Medicine and Public Health, University of Granada, Granada, Spain

**Keywords:** human papillomavirus, anogenital cancer, epidemiology, incidence, mortality, survival, cervical cancer, time trends

## Abstract

**Introduction:**

HPV infection is a common risk factor for all anogenital cancers. However, there are important differences in the epidemiology of anogenital cancers and these have not been compared considering diverse epidemiological indicators over a long period of time. To fill this gap, we investigated incidence, mortality, and survival trends of anogenital cancers over a period of three decades.

**Methods:**

We conducted an observational registry-based study using data from the population-based cancer registry of Granada in southern Spain. We collected data on all incident cases of anogenital cancer (cervical, anal, penile, vulvar, and vaginal cancer) diagnosed between 1985 and 2017. We calculated crude and age-standardized incidence and mortality rates, and 1, 3, and 5-year overall and net survival. We further conducted time-trend analysis calculating annual percent changes (APC) for each cancer site.

**Results:**

The incidence of anogenital cancers decreased slightly during the past 30 years, with the exception of vulvar cancer, where a slight increase was observed. Mortality decreased significantly for cervical cancer over the study period but increased non-significantly for the remaining cancer sites. Survival rates were similar to those reported in comparable countries and increased for cervical and vulvar cancer.

**Discussion:**

Cervical cancer was the greatest contributor to the burden of anogenital cancers and showed a marked improvement in all indicators in comparison to the remaining cancer sites.

## 1. Introduction

Infection with the human papillomavirus (HPV) is the most common sexually transmitted infection worldwide. Estimates of the prevalence of HPV infection vary widely, suggesting that more than 80% of men and women are likely to be infected at least once during their lifetime (1). The rate of infection peaks in adolescence for women (2) and at later ages for men (3), and varies widely across regions and populations, most likely due to variations in sexual practices (2, 3). The majority of HPV infections are cleared by the immune system without major consequences; however, certain HPV types are oncogenic and can cause a number of different head and neck and anogenital cancers (4).

Since the relationship between the human papillomavirus (HPV) and cancer was first identified in the 1970's, our understanding of the virus and its implications for cancer has grown exponentially (5, 6). Nowadays, HPV is known to cause up to 3.8% of new cancers worldwide (7), most of them occurring in the anogenital area. The most frequent anogenital cancer is cervical cancer, which accounted for an estimated 604,000 new cancer cases and 342,000 deaths in 2020 (8). Other anogenital cancer sites associated with HPV include anal, penile, vaginal, and vulvar cancer, with a burden of 51,000, 36,000, 18,000 and 45,000 new cases worldwide in 2020, respectively (8). HPV infection is a common risk factor for anogenital cancers, however, there are important differences in the epidemiology of the different anogenital sites.

Cervical cancer is the only HPV-related cancer for which there are currently effective screening tests in place. Following the implementation of cervical screening programs, the incidence of cervical cancer in Europe (with the exception of certain countries in Eastern Europe) has notably declined (9). Nevertheless, an upward incidence trend in young European women has been described, which may be due to the high prevalence of high-risk HPV genotypes, the evolution of sexual practices, and inadequate screening uptake (10). In Spain, the incidence and mortality rates of cervical cancer have stabilized in recent years, with age-standardized rates below the average in Europe (11).

Anal cancer has been on the rise over the last decades, particularly among men. Certain sexual practices, the number of sexual partners, frequency of receptive anal sex, and HIV infection have been found to increase the risk of exposure to high-risk HPV genotypes (12). The incidence of anal cancer is expected to further grow worldwide within the next years (13), especially in high-risk groups such as HIV-positive men who have sex with men, who have annual incidence rates above 131 cases per 100,000 men, and in women with a previous cancer caused by HPV (14, 15). In contrast, in Spain, anal cancer trends have remained stable (16).

In 2020, there were 36,000 new penile cancer cases worldwide and 13,000 deaths due to penile cancer (8). The main risk factors for this cancer include phimosis, lack of hygiene, and other factors also involved in cervical and anal cancer (17). Despite its relatively low incidence, a recent study from the US has shown an increasing trend (18), whereas mortality might be slightly decreasing according to evidence from Germany (19).

With respect to vulvar cancer, its incidence is growing in Western countries (20). This phenomenon, mainly observed in younger age groups, could be explained by the frequency of HPV infection: a 15% absolute increase was reported in women below 60 years in Europe between 1988 and 2007 (21).

Vaginal cancer is responsible for almost 18,000 new cases and 8,000 deaths annually worldwide (8). A recent population-based study from Denmark showed that its incidence has followed a downward trend after the introduction of vaccines against HPV (22).

Survival for anogenital cancers is also variable and strongly dependent on stage at diagnosis. Relative 5-year survival rates for all stages combined tend to be between 65% and 70% for the majority of anogenital cancers (67% for cervical, 70% for anal, 65% for penile, 70% for vulvar), with the exception of vaginal cancer, which has worse survival (49%) (23, 24).

Anogenital cancers share common etiology, however, they are rarely studied as a group and considering different epidemiological indicators simultaneously over a long period of time. Previous studies have either focused on specific cancer sites (19) or have compared different anogenital cancers on a specific epidemiological indicator such as incidence (15). There are no recent studies reporting incidence, mortality, and survival trends for the different anogenital cancers. To fill this gap and improve our understanding of the epidemiology of anogenital cancers, we conducted a population-based study of incidence, mortality, and survival trends of cervical, anal, penile, vulvar, and vaginal cancer over a period of three decades. In particular, we conducted an observational registry-based study using data for the province of Granada in southern Spain and covering the period between 1985 and 2017.

## 2. Methods

### 2.1. Study participants and data sources

We collected data on all incident cases of anogenital cancer in Granada diagnosed between 1985 and 2017. The following ICD-O-3 cancer sites were considered: anus (C21), vulva (C51), vagina (C52), cervix uteri (C53), and penis (C60).

Incidence data were obtained from the Granada Cancer Registry (GCR), a population-based cancer registry in Southern Spain that began its activity in 1985, currently covering a population of 930,000 inhabitants (50.5% women). GCR information sources include clinical records from public and private hospitals, files from Anatomical Pathology and Oncology units, and death certificates. Data from the GCR are regularly published in the IARC monographs “Cancer Incidence in Five Continents” (25). The GCR is a member of the Spanish Network of Cancer Registries (REDECAN) and the European Network of Cancer Registries (ENCR). It collaborates in the international projects EUROCARE (http://www.eurocare.it) and CONCORD (https://csg.lshtm.ac.uk/research/themes/concord-programme/). In terms of data quality, 96.8% of cancers in the anogenital area had pathological confirmation (by histology or cytology) and the death certificates constituted the sole information source in only 0.7% of cases.

Follow-up of cases was conducted using a mixed method, through linkage with the National Index of Mortality (Indice Nacional de Defunciones) and through active case finding (i.e., revision of clinical records) of cancer cases with poor prognosis and advanced age. Time of follow-up was defined as the time interval elapsed between the date of diagnosis and the date of death (for deceased patients), the date of last contact recorded (for patients lost at follow-up) or 31 December, 2021 (for the rest). Mortality data were retrieved from the information system of the Spanish Ministry of Health, considering the same ICD-O-3 codes and study period employed for incidence data.

### 2.2. Study variables

To study the incidence of anogenital cancers, the following variables were included: age at diagnosis, sex, anatomical site, tumor morphology and behavior according to ICD-O-3, most valid data source for diagnosis, date of diagnosis, date of last contact recorded, and vital status. Regarding mortality, age group, sex, ICD-10 anatomical site and year of death were included.

Age at diagnosis was categorized in 10-year intervals for the specific analysis: <25, 25–34, 35–44, 44–54, 55–64 and ≥65 years. The study period was divided into three time periods: from 1985 to 1997, from 1998 to 2007, and from 2008 to 2017, as we hypothesized that sexual behaviors—which influence the risk of HPV infection –, may have varied over an extended period of time. Additionally, this enabled the estimation of epidemiological indicators for more homogeneous cases of anogenital cancer (hence facilitating inter-period comparisons).

### 2.3. Analysis

The crude and age-standardized incidence rates were estimated by the direct method using the standard European (ASIR-E) and World (ASIR-W) populations. In the analysis as a function of age group, the specific incidence rates were estimated.

In the time-trend analysis, the log-linear joinpoint regression method was applied to the age-standardized incidence rates (ASIR-E) in each year of incidence (26). This technique estimates the annual percent change (APC) and its 95% confidence interval, along with the time points where the trend significantly changes.

Analogous to the incidence study, crude (CMR) and adjusted (ASMR-E and ASMR-W) rates were calculated for mortality data, and a time-trend analysis was also conducted.

We calculated overall survival, which considers all causes of death, and net survival, defined as the survival that would be observed if cancer were the only possible cause of death. Overall survival with 95% confidence intervals was obtained by the Kaplan-Meier method; net survival was obtained using the Pohar-Perme estimator (27), with life tables for competing death probabilities (according to year, sex and age), smoothed by the Elandt-Johnson method (28). In order to draw comparisons between time periods and between sexes, net survival was further standardized by age, utilizing the standard population of patients with cancer (28).

All statistical analyses were performed using Stata software version 17 (StataCorp. 2021. Stata Statistical Software: Release 17. College Station, TX: StataCorp LLC.) with the exception of trend analysis, for which Joinpoint software version 4.9 was used (Joinpoint Regression Program, Version 4.9.1.0–April 2022; Statistical Methodology and Applications Branch, Surveillance Research Program, National Cancer Institute).

## 3. Results

### 3.1. Incidence

**Figure 1:**
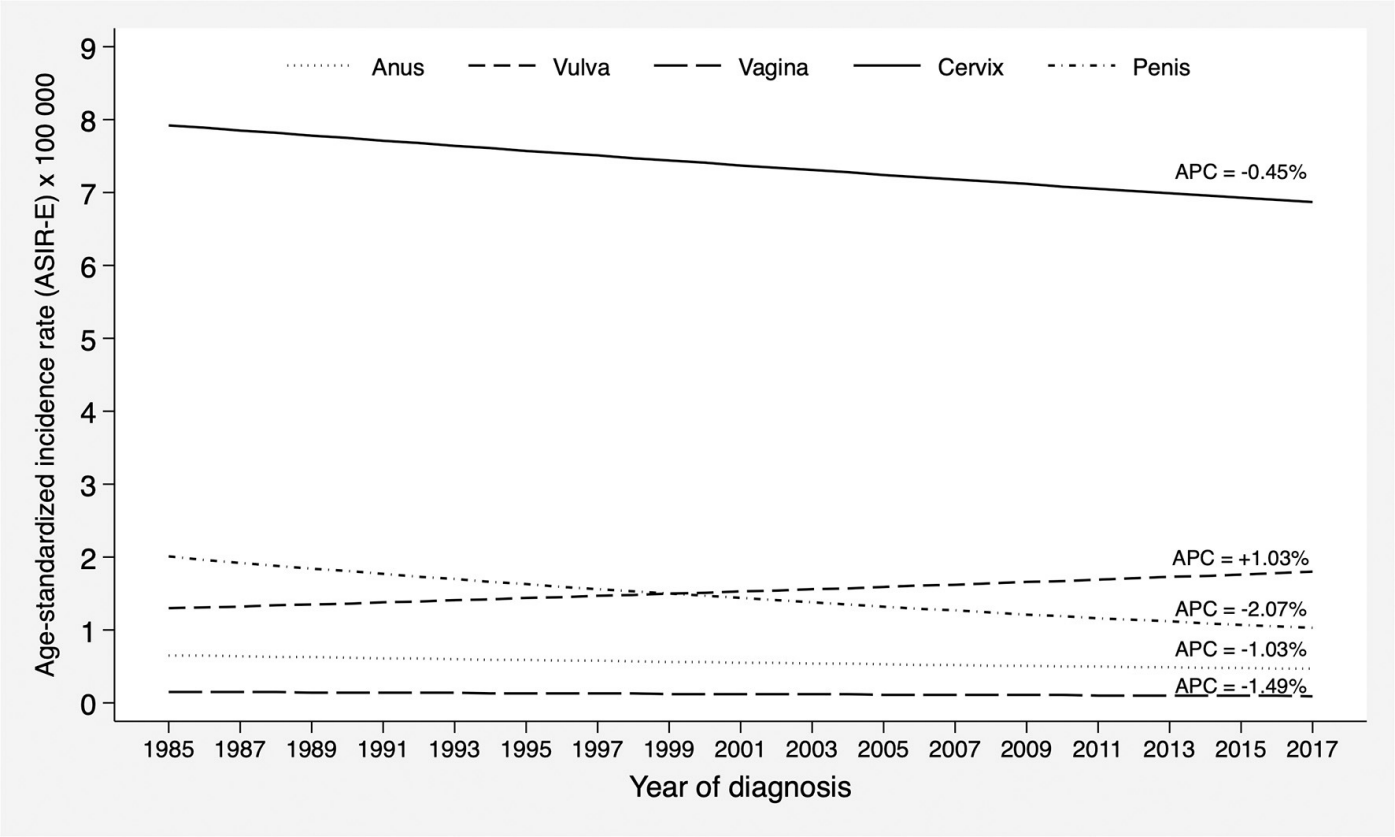
Incidence trends of cancers of the anogenital area in the province of Granada in the period 1985–2017. APC, Annual Percent Change.

Between 1985 and 2017, a total of 1,951 cases of cancer of the anogenital area were registered. These included 1,112 cases of cervical cancer, 205 cases of anal cancer, 234 cases of penile cancer, 343 cases of vulvar cancer, and 57 cases of vaginal cancer (see Table 1). The trend analysis of the standardized incidence ratio (ASIR-E) showed no statistically significant changes for any cancer site during the study period (see Figure 1). The APC was overall negative, with some variation according to anatomical site. For cervical cancer, there was a slightly decreasing trend (APC = −0.45%; 95% CI: −1.2 to 0.3), similar to that of anal cancer (APC = −1.03%; 95% CI: −2.8 to 0.8), penile cancer (APC = −2.07%; 95% CI: −4.3 to 0.2), and vaginal cancer (APC = −1.49%; 95% CI: −9.2 to 6.9); in contrast, an increasing trend was observed for vulvar cancer (APC = 1.03%; 95% CI: −0.4 to 2.5). Were these trends to continue, the estimated ASIR-E of anogenital cancers by 2022 would be 1.7 per 100,000 men and 8.0 per 100,000 women (Supplementary Figure S1).

**Table 1:** Incidence of cancers of the anogenital area in the province of Granada from 1985 to 2017.

	**1985–1997**				**1998–2007**				**2008–2017**			
	**Cases**	**CIR**	**ASIR-E**	**ASIR-W**	**Cases**	**CIR**	**ASIR-E**	**ASIR-W**	**Cases**	**CIR**	**ASIR-E**	**ASIR-W**
Cervix uteri	400	7.60	7.79	5.9	334	7.79	7.37	5.72	378	8.17	7.33	5.72
Anus^*^	76	0.74	0.69	0.47	56	0.67	0.54	0.36	73	0.80	0.59	0.41
Penis	82	1.62	1.77	1.17	68	1.65	1.54	1.08	84	1.85	1.46	0.97
Vulva	102	1.94	1.54	1.01	106	2.47	1.62	1.08	135	2.92	1.74	1.11
Vagina	19	0.36	0.31	0.22	21	0.49	0.37	0.24	17	0.37	0.22	0.14
Total^*^	679	6.58	6.63	4.78	585	6.95	6.17	4.53	687	7.49	6.04	4.42

CIR, Crude Incidence Rate; ASIR-E, age-standardized rate referred to European population; ASIR-W, age-standardized rate referred to world population. Rates per 100,000 inhabitants. ^*^Both sexes.

Overall, four out of five incident cancers of the anogenital area (82.1%) occurred in females, as cervical cancer was the most frequent malignancy, responsible for 57.0% of all cases. The second most frequent cancer was vulvar cancer, and the least frequent was vaginal cancer (2.9%). In men, penile cancer was the most frequent with 234 cases (12.0%), followed by anal cancer (5.4%).

**Figure 2:**
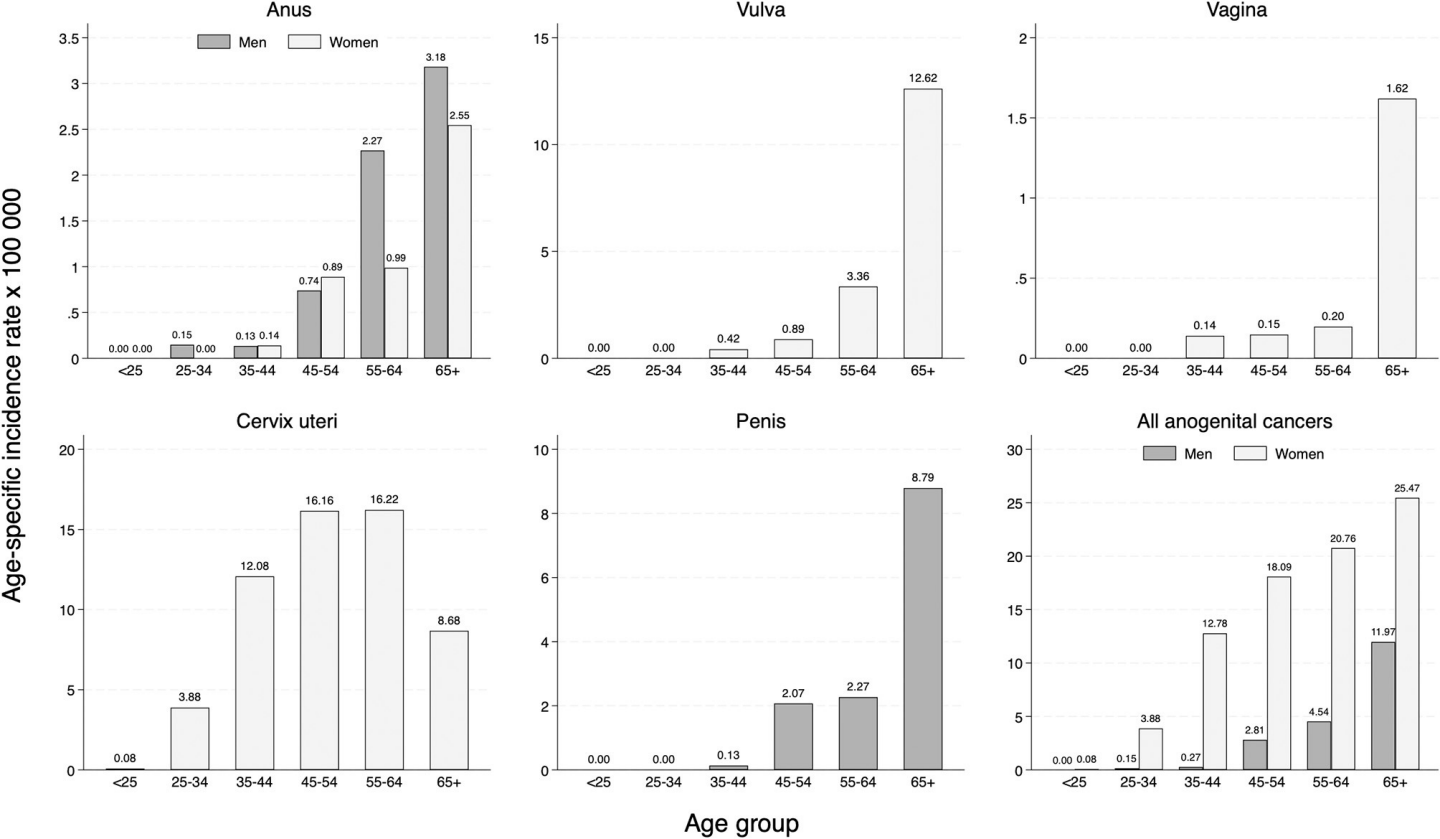
Age-specific incidence rate of cancers of the anogenital area in the province of Granada (period 2008–2017).

Higher incidence was found for individuals ≥65 years old for all cancer sites (irrespective of sex). The one exception was cervical cancer, where the highest incidence occurred in women 45–64 years old (see Figure 2). More than 90% of vulvar cancers were diagnosed in women aged 55 years and above, and most vaginal cancers also occurred in women ≥65 years old. Likewise, two thirds of cases of penile cancer occurred in men ≥65 years old. With regard to anal cancer, men were more frequently affected (53.4%) compared to women, particularly those aged 55 years and over; for both sexes, incidence increased with age. In terms of the spatial distribution, the ASIR-E of anogenital cancers between 2008 and 2017 ranged from 9.3 to 10.5 per 100,000 women and from 1.9 to 2.5 per 100,000 men across health districts (Supplementary Figure S2). Of note, the Granada district (which includes the province's capital city) showed the highest ASIR-E in women and the second highest ASIR-E in men; at any rate, the variability observed was quite low.

### 3.2. Mortality

During the study period, there were 532 fatalities caused by the anogenital cancers studied: 311 were due to cervical cancer (58.5%), followed by vulvar (22.4%), and penile cancer (8.1%) (see Table 2). Similar to the sex distribution of incident cancers, women accounted for 88% of the deaths registered.

**Table 2:** Mortality from cancers of the anogenital area in the province of Granada from 1985 to 2017.

	**1985–1997**				**1998–2007**				**2008–2017**			
	**Deaths**	**CMR**	**ASMR-E**	**ASMR-W**	**Deaths**	**CMR**	**ASMR-E**	**ASMR-W**	**Deaths**	**CMR**	**ASMR-E**	**ASMR-W**
Cervix uteri	126	2.39	2.26	1.61	99	2.31	2.03	1.49	86	1.86	1.43	1.05
Anus^*^	8	0.08	0.06	0.04	14	0.17	0.14	0.10	11	0.12	0.09	0.06
Penis	15	0.30	0.33	0.21	10	0.24	0.21	0.13	18	0.40	0.31	0.20
Vulva	35	0.66	0.52	0.33	45	1.05	0.59	0.34	39	0.84	0.43	0.27
Vagina	8	0.15	0.13	0.10	6	0.14	0.09	0.05	12	0.26	0.16	0.09
Total^*^	192	1.86	1.80	1.23	174	2.07	1.71	1.16	166	1.81	1.30	0.90

CMR, Crude Mortality Rate; ASMR-E, age-standardized rate referred to European population; ASMR-W, age-standardized rate referred to world population. Rates for 100,000 inhabitants. ^*^Both sexes.

**Figure 3:**
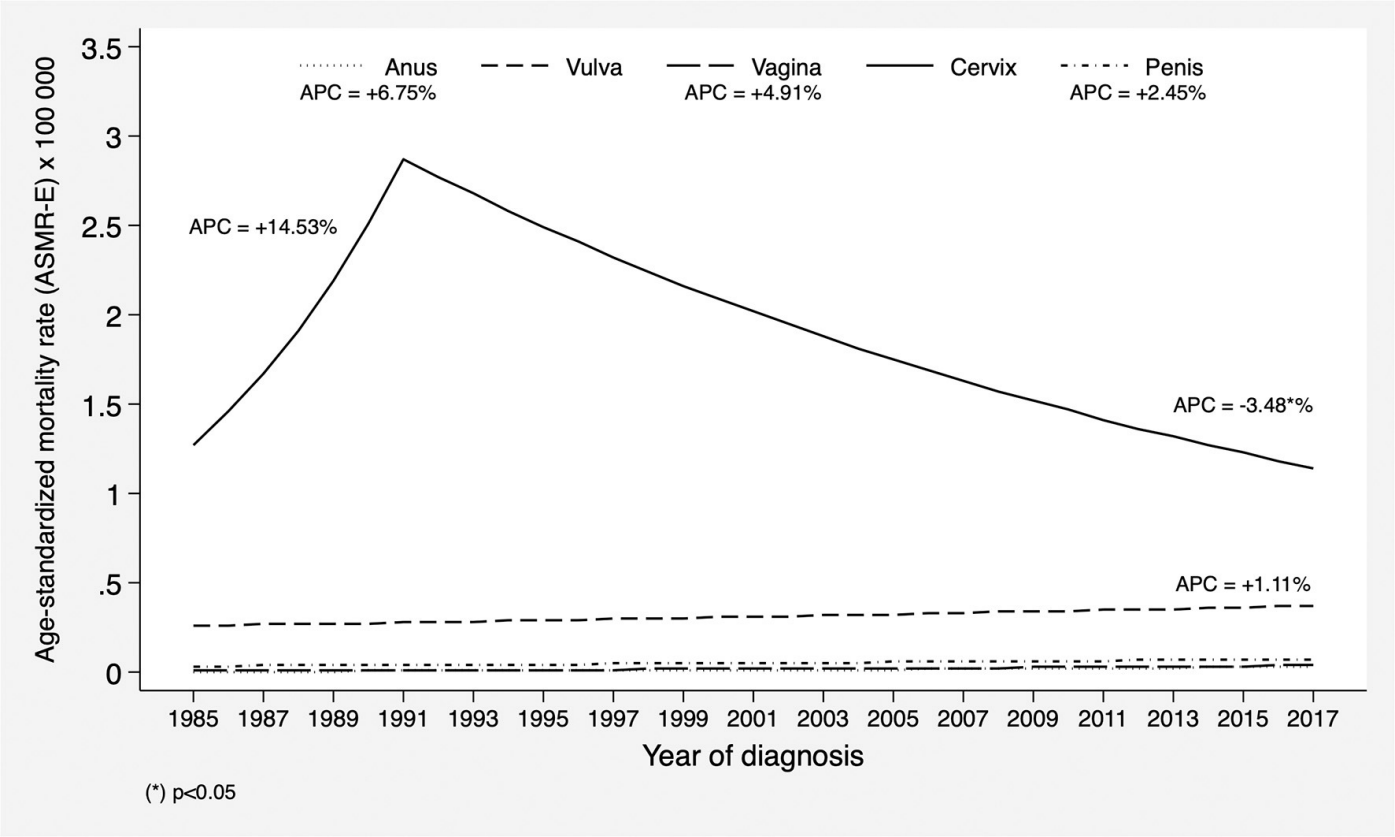
Mortality trends of cancers of the anogenital area in the province of Granada in the period 1985–2017. APC, Annual Percent Change.

Figure 3 shows the trend analysis of the standardized mortality rates (ASMR-E), which yielded a statistically significant decreasing trend for cervical cancer, going down from 2.26 in the first time interval (1985–1997) to 1.43 in the third (2008–2017). This resulted in a statistically significant APC of −3.48% (95% CI: −5.1 to −1.8) from 1991 to 2017 (an accused growing trend was observed prior to 1991). For the remaining cancer sites, the APC trend in the study period was increasing, albeit not significantly.

**Figure 4:**
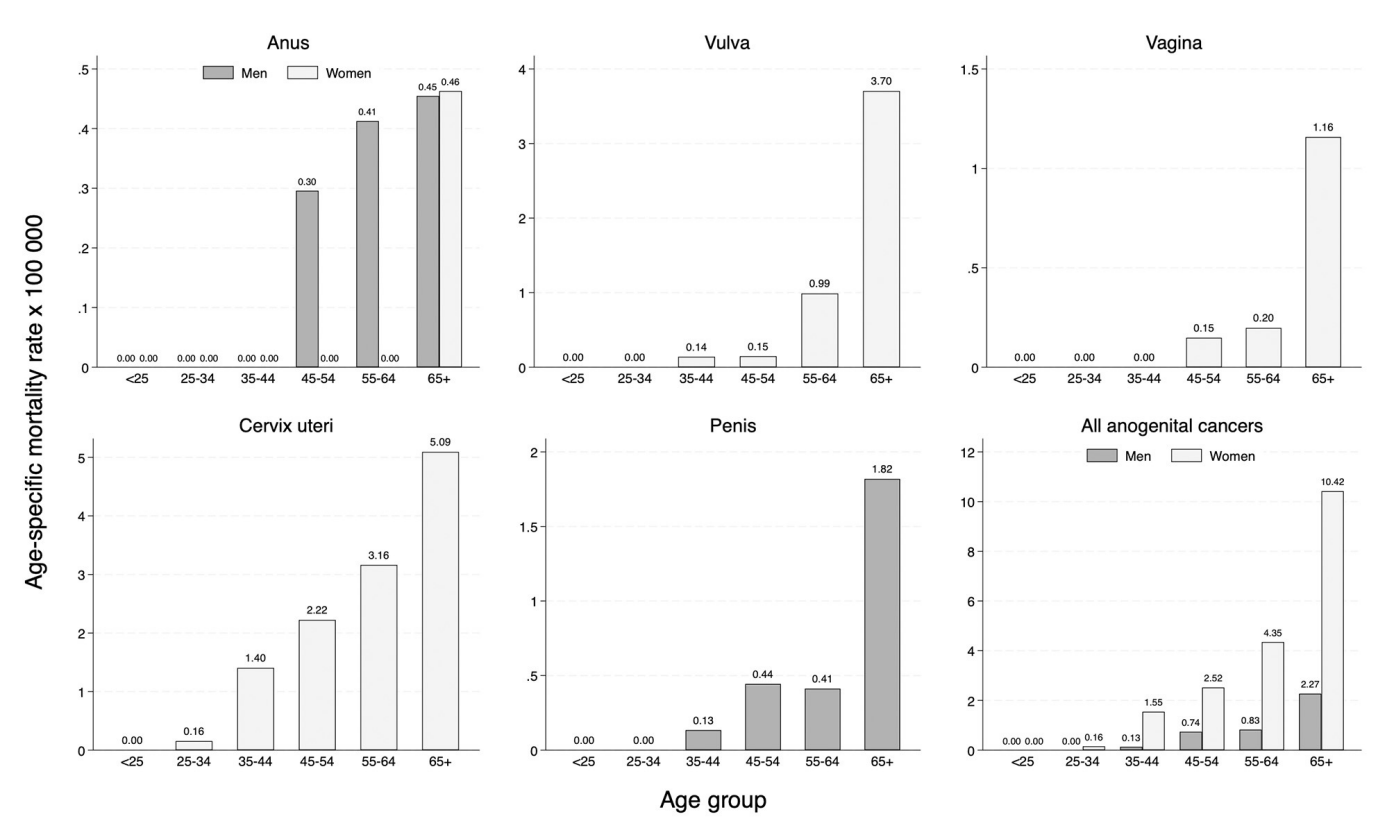
Age-specific mortality rate of cancers of the anogenital area in the province of Granada (period 2008–2017).

Mortality globally increased with more advanced age for all cancer sites (see Figure 4). Women of advanced age, particularly above 65 years, had higher mortality for cervical, vaginal, and vulvar cancer (Figure 4). In women, anal cancer mortality was higher among those ≥65 years (specific mortality rate = 0.46); in contrast, in men, age differences in anal cancer mortality were much smaller.

### 3.3. Survival

**Figure 5:**
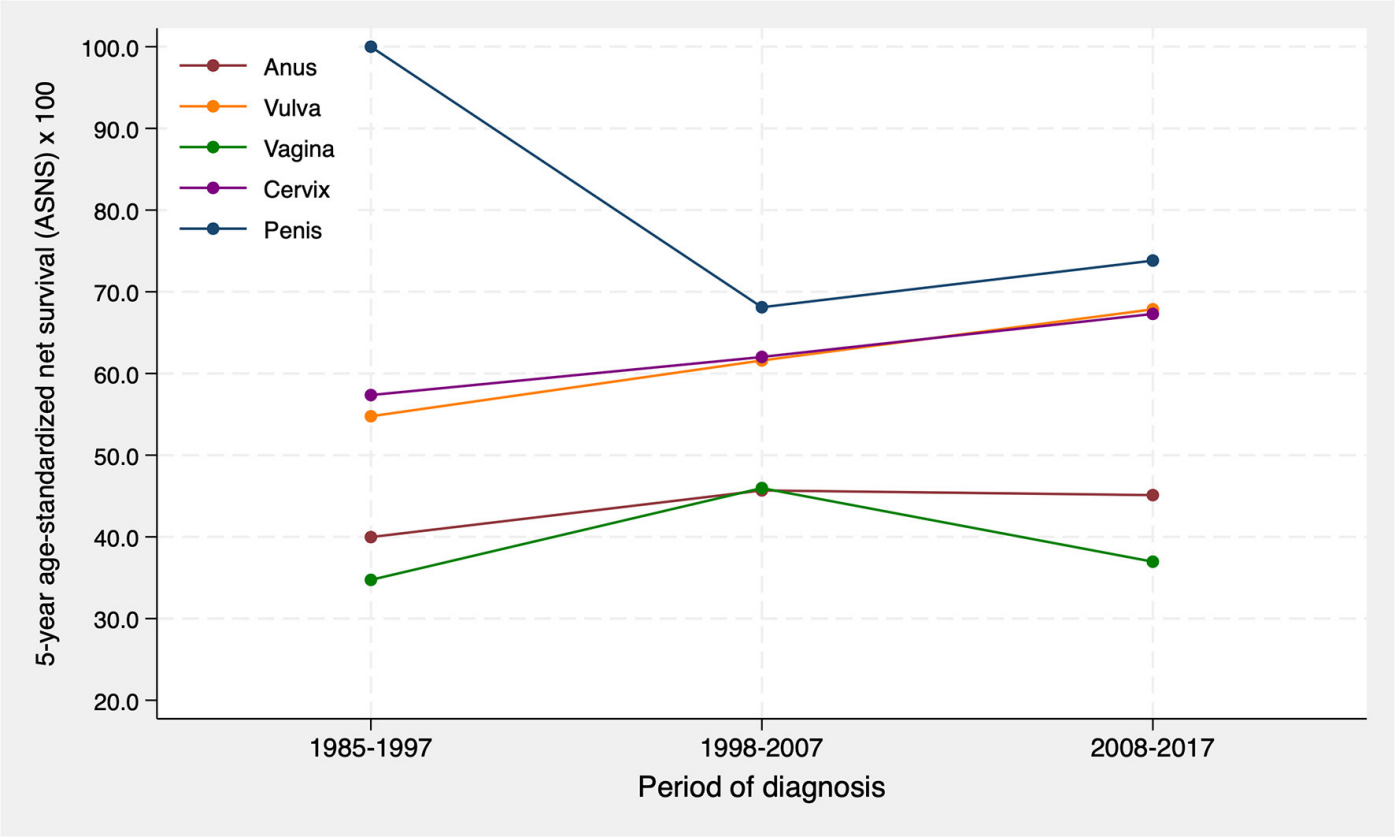
5-year age-standardized net survival trends for cancers of the anogenital area in the province of Granada during the period 1985–2017.

As illustrated in Figure 5, cervical and vulvar cancer showed an upward trend in 5-year survival between 1985 and 1997 and 2008–2017, with a 9.9% increase (from 57.4% to 67.3%) and a 13.8% increase (from 54.1% to 67.3%), respectively. For penile cancer an irregular evolution was observed, with an estimated 5-year survival of 68% in the second period compared to 100% in the first period, and then increasing to 74% in the last period. However, no statistically significant changes were observed for any cancer site.

The 1-year, 3-year, and 5-year survival rates (including overall survival, net survival, and age-standardized net survival) in the period 2008–2017 for all cancer sites studied are displayed in Table 3. Anal cancer survival rates stratified by sex are further detailed in Supplementary Table S1.

**Table 3:** 1, 3 and 5-year survival for cancers of the anogenital area in the province of Granada for the period 2008–2017.

		**2008-2017**								
		**1-year**			**3-years**			**5-years**		
	**Cases at risk**	**OS**	**NS (95%CI)**	**ASNS (95%CI)**	**OS**	**NS (95%CI)**	**ASNS (95%CI)**	**OS**	**NS (95%CI)**	**ASNS (95%CI)**
Cervix uteri	377	87	88 (84–91)	86 (81–89)	74	75 (70–79)	71 (66–76)	70	71 (66–76)	67 (62–72)
Anus^*^	73	67	69 (56–78)	70 (58–79)	47	49 (36–61)	51 (39–63)	38	42 (29–55)	45 (32–58)
Penis	84	83	87 (75–93)	89 (80–94)	68	76 (62–86)	75 (64–83)	64	76 (56–88)	74 (59–84)
Vulva	135	72	74 (65–81)	82 (75–88)	58	64 (54–72)	69 (58–77)	53	64 (53–74)	68 (56–77)
Vagina	16	69	71 (41–88)	73 (51–86)	38	41 (16–65)	53 (42–62)	25	32 (9–57)	37 (15–60)
Total^*^	685	81	82 (79–85)	80 (77–83)	66	69 (65–73)	66 (62–70)	61	66 (62–70)	63 (58–68)

OS, overall survival; NS, Net survival; ASNS, age-standardized net survival. Probabilities in percent (%). ^*^Both sexes.

## 4. Discussion

To the best of our knowledge, this is the first population-based study to offer a long-term overview of the trends in incidence, mortality, and survival of anogenital cancers. We found relatively stable incidence trends, with slight non-significant decreases in cervical, anal, penile, and vaginal cancer, and a slight non-significant increase in vulvar cancer. Cervical cancer continues to be the most frequent cancer, representing 55% of all anogenital cancers. There was also a significant reduction in mortality from cervical cancer over the study period. In contrast, mortality for the other cancer sites showed a slight non-significant increase. Survival increased non-significantly for cervical and vulvar cancer.

Women had an incidence of anogenital cancers 4 times greater than that of men, with 82% of cases and 88% of deaths occurring in women. Sex differences reported for these HPV-related cancer sites in Canada were even sharper (29). This is mainly due to the contribution of cervical cancer to the total number of anogenital cancers (55% in the current study), similar to the proportion reported in a study conducted in Scotland (52%) (30). Other studies from high-income countries have found that cervical cancer may account for up to two thirds of all anogenital cancers (29, 31). The relative contribution of the remaining cancer sites to the total number of anogenital cancer cases remained stable during the study period. In descending order, these were vulvar, penile, anal, and vaginal cancer.

### 4.1. Cervical cancer

In the current study, half of all cervical cancers affected women aged 45–64 years, consistent with data from other population-based cancer registries (31, 32). An overall decline was observed in cervical cancer incidence since 1985, but not as marked as the trends previously described in Spain (33, 34), the United States (35), China (36), and Poland (37). This discrepancy may stem from the low regional incidence of cervical cancer at the start of the study in comparison to other geographical locations (32, 37). Another potential factor is compliance with the cervical cancer screening program, formally included in the Spanish National Health System in 2014 (38); Andalusia, the autonomous community to which Granada belongs, is among those with poorer compliance (39). In fact, according to the incidence rates obtained between 2008 and 2017, Granada would currently present an incidence above the average in Spain (25).

The increasing trend in mortality observed during the first six years of the study period could be due to the absence of cervical cancer screening programs and the lower accuracy of imaging tests, which led to diagnosis in more advanced stages; and by the limited effectiveness of the treatment available at the time. Since then, Bosetti et al. (40) identified the decline in cervical cancer mortality as one of the major contributors in the reduction of cancer mortality in Europe. We found a decreasing trend for cervical cancer mortality, reaching an ASMR-E = 1.43 deaths per 100,000 women between 2008 and 2017, similar to the values reported in Northern and Western Europe (11, 32, 41). This finding may be explained by cervical cancer screening, which has shown to decrease cervical cancer mortality in Europe (42), and by the inclusion of chemotherapy in the therapeutic arsenal for advanced cervical cancer (43). Moreover, immunotherapy might further reduce cervical cancer mortality in upcoming years (44). The 5-year age-standardized net survival (taking the European population as reference) was estimated at 67%, in accordance with the rates reported in other studies (30, 33, 45).

### 4.2. Vulvar cancer

Vulvar cancer represented between 15% and 20% (in 1985–1997 and 2008–2017, respectively) of all anogenital cancers. A smaller contribution, usually around 10%, has been found in similar studies (29, 46, 47). The incidence of vulvar cancer found in our study is comparable to that found in other high-income countries, although slightly higher than the incidence reported in studies from Denmark and South Korea (46, 48) and slightly lower than in studies from Germany and Japan (49, 50). Most vulvar cancers were diagnosed in women ≥65 years old, consistent with findings from other populations (51, 52).

Vulvar cancer was also ranked second in terms of mortality and the ASMR-E in our population was lower than the one found in other European countries (53). It is possible that prognostic factors as the tumor stage at diagnosis (not included in our study) underlie these differences. There was little variation in survival throughout the period analyzed, with a 5-year ASNS of 64%, concordant with global estimates (52) and with the period-specific survival rates recently described in the United States (54).

### 4.3. Vaginal cancer

The anogenital cancer with the lowest incidence and mortality was vaginal cancer; yet, its mortality increased, especially during the last decade analyzed. The incidence and mortality rates observed are very similar to those found in Denmark (46). However, both higher incidence (30) and higher mortality (as much as 3-fold, with an ASMR-W of 0.3 per 100,000 women) (48) have been reported for other countries. Almost all cases occurred in women older than 65 and this was the age group with highest mortality, similar to previous studies (22). The survival rates for vaginal cancer in our population are below those reported in other countries (54). Stage at diagnosis, which was not included in the survival analysis, may account for these differences.

### 4.4. Anal cancer

The epidemiological indicators for anal cancer showed some differences with previous research. Anal cancer ranked fourth in terms of incidence, in contrast to studies from other high-income countries that have found it is the second most frequent anogenital cancer (46, 47, 55). Studies from several other countries have reported higher age-standardized incidence rates than those found in the current study (30, 31) and rising incidence trends (56), something that was not observed in our study. We found the highest incidence in the oldest age groups, whereas several studies from other countries have reported highest incidence for individuals aged 45 to 69 (15, 57). In our study, anal cancer was more frequent in men than in women, as described in previous studies analyzing nationwide data (17, 25).

Almost two thirds of deaths from anal cancer occurred in men and mortality increased with age. Mortality followed an upward trend, but was still well below rates at the national (17) and global level (58). With respect to survival, the 5-year survival rates observed in this study were lower than in comparable populations (54, 59). Survival rates, which are influenced by stage at diagnosis and type of treatment, among others, may vary between 80% in localized disease and 20% in metastatic cancer.

### 4.5. Penile cancer

We found higher incidence rates for penile cancer compared to North America (47, 55). We also observed a slight decrease in incidence, in contrast to other studies showing stable or increasing temporal trends (19, 56, 60). However, the incidence rates obtained (ASIR-E and ASIR-W) were similar to those found in cancer registries in other European countries, that have a similar prevalence of circumcision (25, 61). We also found that penile cancer mortality increased during the study period, with rates concordant with data from other countries (19, 61). Penis was the cancer site with second highest survival between 2008 and 2017: its 5-year survival rate exceeded 70%, similar to values reported for countries from Northern Europe (62).

### 4.6. Epidemiology of HPV infection

HPV infection is one of the most frequent sexually-transmitted infection worldwide (63). The prevalence of genital HPV infection shows great variation across populations, ranging from 2% to 45%, and is higher in less-developed regions, namely in Oceania and Africa (64). Such large differences are related to several risk and protective factors that we now briefly summarize.

Subjects with a weakened immune system are more susceptible to HPV infection. In particular, those infected by human immunodeficiency virus (HIV) or undergoing immunosuppressive therapies (for instance, in the context of solid-organ transplantation) are at higher risk (65). Conversely, vaccination against HPV, especially with the 9-valent vaccine, is highly effective at reducing the incidence of HPV infection (66). Besides, tobacco smoking has also shown to increase the risk of acquiring HPV (67). Furthermore, psychosocial aspects may be even more relevant. Since HPV is primarily transmitted through sexual contact, factors such as early age at sexual debut, high number of sexual partners, risky sexual behaviors, and measures to improve risk perception (for instance, by providing adequate sexual education to young and adolescent individuals) or lack thereof all influence the risk of exposure to HPV and of a subsequent potential infection (68).

Many of the abovementioned factors show an uneven global distribution. HIV infection still disproportionately affects sub-Saharan Africa; about 15% of girls in the target age for HPV vaccination are fully immunized (69); the overall burden of tobacco consumption is mainly carried by low- and middle-income countries (70); multiple countries have room for improvement in terms of education (71), let alone sexual education.

### 4.7. Limitations

To the best of our knowledge, this is the first study to simultaneously analyze incidence, mortality and survival trends for all anogenital cancer sites over a period longer than 30 years. However, it presents some limitations. Firstly, with the exception of cervical cancer, the cancers studied are relatively rare. In addition, the population from which the Granada Cancer Registry draws data is also relatively small (930,000 inhabitants). As a result, the number of cases is generally insufficient to reach high statistical power, increasing the probability of type 2 errors (failing to find significant differences when these actually exist). Secondly, multiple factors involved in the etiology of anogenital cancer, such as the prevalence of HPV infection, a previous history of anogenital cancer, and sociocultural determinants, were not included. Although vaccination against HPV may influence epidemiological data, we believe that it has not affected our results, because it was not included in the regional systematic vaccination schedule until 2008 (when it was also restricted to females aged 14 years) and the period elapsed between HPV infection and anogenital cancer development can range from years to several decades. Thirdly, while this study covers five different cancer types, the epidemiology of HPV infection is not limited to the anogenital area—HPV has also been involved in the development of other cancers, namely oropharyngeal cancer (72). Consequently, the total contribution of this pathogen to the cancer burden is to some extent greater than presented here. However, the decision to restrict the cancer sites analyzed to those in the anogenital area increased the comparability of our estimates. Finally, we were unable to adjust for key prognostic factors like the tumor stage at diagnosis or the type of treatment.

## 5. Conclusions

This population-based study in southern Spain offered estimates of the incidence, mortality, and survival trends for anogenital cancers during more than three decades. The incidence of anogenital cancers decreased slightly during the past 30 years, with the exception of vulvar cancer, where a slight increase was observed. Mortality decreased significantly for cervical cancer over the study period but increased non-significantly for the remaining cancer sites. Survival rates were similar to those reported in comparable countries and increased for cervical and vulvar cancer. Cervical cancer was the greatest contributor to the burden of anogenital cancers and showed a marked improvement in all indicators in comparison to the remaining cancer sites. Vaccination against HPV is likely to have an impact on the incidence, mortality and survival of HPV-related anogenital cancers and should be addressed in future studies using data from population-based registries.

## Data availability statement

The data analyzed in this study is subject to the following licenses/restrictions: Population-based Spanish Cancer Registries are only allowed to publish data in aggregate form. Individualized data can be requested from the Cancer Registry of Granada via the corresponding author or the following webpage: https://www.registrocancergranada.es. Data requestors will need to sign a data access agreement. Requests to access these datasets should be directed to M-JS, mariajose.sanchez.easp@juntadeandalucia.es.

## Ethics statement

Ethical approval was not required for the study involving humans in accordance with the local legislation and institutional requirements. Written informed consent to participate in this study was not required from the participants or the participants' legal guardians/next of kin in accordance with the national legislation and the institutional requirements.

## Author contributions

M-JS and JG contributed to conception and design of the study. MR-B organized the databases and performed the statistical analysis. PD-L, NF-M, and DP wrote the first version of the manuscript. JJ-M critically reviewed the manuscript. All authors contributed to manuscript revision, read, and approved the submitted version.

## References

[1] Chesson HW Dunne EF Hariri S Markowitz LE. The estimated lifetime probability of acquiring human papillomavirus in the United States. Sex Transm Dis. (2014) 41:660–4. 10.1097/OLQ.0000000000000193 25299412 PMC6745688

[2] Smith JS Melendy A Rana RK Pimenta JM. Age-specific prevalence of infection with human papillomavirus in females: a global review. J Adolesc Health. (2008) 43:S5.e1–2. 10.1016/J.JADOHEALTH.2008.07.009 18809145

[3] Smith JS Gilbert PA Melendy A Rana RK Pimenta JM. Age-specific prevalence of human papillomavirus infection in males: a global review. J Adolesc Health. (2011) 48:540–52. 10.1016/J.JADOHEALTH.2011.03.010 21575812

[4] Brianti P De Flammineis E Mercuri SR. Review of HPV-related diseases and cancers. New Microbiol. (2017) 40:80–5. 28368072

[5] zur Hausen H. Human papillomaviruses and their possible role in squamous cell carcinomas. Curr Top Microbiol Immunol. (1977) 78:1–30. 10.1007/978-3-642-66800-5_1 202434

[6] Zur Hausen H. Papillomaviruses and cancer: from basic studies to clinical application. Nat Rev Cancer. (2002) 2:342–50. 10.1038/NRC798 12044010

[7] de Martel C Georges D Bray F Ferlay J Clifford GM. Global burden of cancer attributable to infections in 2018: a worldwide incidence analysis. Lancet Glob Heal. (2020) 8:e180–90. 10.1016/S2214-109X(19)30488-7 31862245

[8] Sung H Ferlay J Siegel RL Laversanne M Soerjomataram I Jemal A. Global cancer statistics 2020: GLOBOCAN estimates of incidence and mortality worldwide for 36 cancers in 185 countries. CA Cancer J Clin. (2021) 71:209–49. 10.3322/CAAC.21660 33538338

[9] Bray F Loos AH McCarron P Weiderpass E Arbyn M Møller H. Trends in cervical squamous cell carcinoma incidence in 13 European countries: changing risk and the effects of screening. Cancer Epidemiol Biomarkers Prev. (2005) 14:677–86. 10.1158/1055-9965.EPI-04-0569 15767349

[10] Torre LA Siegel RL Ward EM Jemal A. Global cancer incidence and mortality rates and trends—An update. Cancer Epidemiol Biomarkers Prev. (2016) 25:16–27. 10.1158/1055-9965.EPI-15-0578 26667886

[11] Arbyn M Weiderpass E Bruni L de Sanjosé S Saraiya M Ferlay J. Estimates of incidence and mortality of cervical cancer in 2018: a worldwide analysis. Lancet Glob Heal. (2020) 8:e191–203. 10.1016/S2214-109X(19)30482-6 31812369 PMC7025157

[12] Gustafsson L Pontén J Bergström R Adami H-O. International incidence rates of invasive cervical cancer before cytological screening. Int J Cancer. (1997) 71:159–65. 10.1002/(SICI)1097-0215(19970410)71:2 9139836

[13] Smittenaar CR Petersen KA Stewart K Moitt N. Cancer incidence and mortality projections in the UK until 2035. Br J Cancer. (2016) 115:1147–55. 10.1038/BJC.2016.304 27727232 PMC5117795

[14] Albuquerque A. Cytology in anal cancer screening: practical review for clinicians. Acta Cytol. (2020) 64:281–7. 10.1159/000502881 31533094

[15] Silverberg MJ Lau B Justice AC Engels E Gill MJ Goedert JJ. Risk of anal cancer in HIV-infected and HIV-uninfected individuals in North America. Clin Infect Dis. (2012) 54:1026–34. 10.1093/CID/CIR1012 22291097 PMC3297645

[16] Islami F Ferlay J Lortet-Tieulent J Bray F Jemal A. International trends in anal cancer incidence rates. Int J Epidemiol. (2017) 46:924–38. 10.1093/IJE/DYW276 27789668

[17] Gil-Prieto R Ester PV Álvaro-Meca A Rodríguez MSM De Miguel ÁG. The burden of hospitalizations for anus and penis neoplasm in Spain (1997–2008). Hum Vaccin Immunother. (2012) 8:201–7. 10.4161/HV.18388 22426377

[18] Chipollini J Chaing S Peyton CC Sharma P Kidd LC Giuliano AR. National trends and predictors of locally advanced penile cancer in the United States (1998–2012). Clin Genitourin Cancer. (2018) 16:e121–7. 10.1016/J.CLGC.2017.07.031 28866244

[19] Schoffer O Neumann A Stabenow R Schülein S Böhm WD Gonsior A. Penile cancer—Incidence, mortality, and survival in Saxony, Germany. Urol Oncol. (2019) 37:295.e1–8. 10.1016/J.UROLONC.2018.12.003 30595462

[20] Akhtar-Danesh N Elit L Lytwyn A. Trends in incidence and survival of women with invasive vulvar cancer in the United States and Canada: a population-based study. Gynecol Oncol. (2014) 134:314–8. 10.1016/J.YGYNO.2014.05.014 24875124

[21] Kang YJ Smith M Barlow E Coffey K Hacker N Canfell K. Vulvar cancer in high-income countries: increasing burden of disease. Int J Cancer. (2017) 141:2174–86. 10.1002/IJC.30900 28730615

[22] Bertoli HK Baandrup L Aalborg GL Kjaer AK Thomsen LT Kjaer SK. Time trends in the incidence and survival of vaginal squamous cell carcinoma and high-grade vaginal intraepithelial neoplasia in Denmark—A nationwide population-based study. Gynecol Oncol. (2020) 158:734–9. 10.1016/J.YGYNO.2020.05.683 32571683

[23] Guevara M Molinuevo A Salmerón D Marcos-Gragera R Carulla M Chirlaque MD. Cancer survival in adults in Spain: a population-based study of the spanish network of cancer registries (REDECAN). Cancers. (2022) 14:441. 10.3390/CANCERS14102441 35626046 PMC9139549

[24] SEER Cancer Statistics Review (CSR). SEER Cancer Statistics. (2021). Available online at: https://seer.cancer.gov/csr/ (accessed February 14, 2023).

[25] Bray F Colombet M Mery L Piñeros M Znaor A Zanetti R. Cancer Incidence in Five Continents, Vol. XI Lyon: IARC (2017).

[26] Kim H-J Fay MP Feuer EJ Midthune DN. Permutation tests for joinpoint regression with applications to cancer rates. Stat Med. (2000) 19:335–51. 10.1002/(sici)1097-0258(20000215)19:3<335::aid-sim336>3.0.co;2-z 10649300

[27] Perme MP Stare J Estève J. On estimation in relative survival. Biometrics. (2012) 68:113–20. 10.1111/J.1541-0420.2011.01640.X 21689081

[28] Elandt-Johnson RC Johnson NL. Survival Models and Data Analysis. 1st ed. Hoboken, NJ: Wiley. (1999). 10.1002/9781119011040

[29] Louchini R Goggin P Steben M. The evolution of HPV-related anogenital cancers reported in Quebec—Incidence rates and survival probabilities. Chronic Dis Can. (2008) 28:99–106. 10.24095/HPCDP.28.3.03 18341764

[30] Wakeham K Kavanagh K. The burden of HPV-associated anogenital cancers. Curr Oncol Rep. (2014) 16:1–11. 10.1007/S11912-014-0402-4 25118645

[31] Robinson D Coupland V Møller H. An analysis of temporal and generational trends in the incidence of anal and other HPV-related cancers in Southeast England. Br J Cancer. (2009) 100:527. 10.1038/SJ.BJC.6604871 19156144 PMC2658550

[32] Anaya-Ruiz M Vincent AK Perez-Santos M. Cervical cancer trends in Mexico: incidence, mortality and research output. Asian Pac J Cancer Prev. (2014) 15:8689–92. 10.7314/APJCP.2014.15.20.8689 25374191

[33] Castro Marqueta P Moreno Crespi J Buxó Pujolràs M Cervantes Amat M Pérez Gómez B Marcos Gragera R. Epidemiología del cáncer de cérvix in situ e invasor en la provincia de Girona 1990-2004: incidencia, mortalidad, supervivencia e historial de cribado. Med Clínica. (2011) 136:192–8. 10.1016/j.medcli.2010.07.010 21051058

[34] Pérez-Gómez B Martínez C Navarro C Franch P Galceran J Marcos-Gragera R. The moderate decrease in invasive cervical cancer incidence rates in Spain (1980–2004): limited success of opportunistic screening? Ann Oncol Off J Eur Soc Med Oncol. (2010) 21(Suppl 3):93. 10.1093/ANNONC/MDQ093 20427362

[35] Kurdgelashvili G Dores GM Srour SA Chaturvedi AK Huycke MM Devesa SS. Incidence of potentially HPV-related neoplasms in the United States, 1978–2007. Cancer. (2013) 119:2291. 10.1002/CNCR.27989 23580435 PMC4567689

[36] Lu Y Li P Luo G Liu D Zou H. Cancer attributable to human papillomavirus infection in China: Burden and trends. Cancer. (2020) 126:3719–32. 10.1002/CNCR.32986 32484937

[37] Nowakowski A Wojciechowska U Wieszczy P Cybulski M Kamiński MF Didkowska J. Trends in cervical cancer incidence and mortality in Poland: is there an impact of the introduction of the organised screening? Eur J Epidemiol. (2017) 32:529–32. 10.1007/S10654-017-0291-6 28780640

[38] BOE-A-2014-11444 Orden SSI/2065/2014, de 31 de Octubre, Por La Que Se Modifican Los Anexos I, II y III Del Real Decreto 1030/2006, de 15 De Septiembre. Por El Que Se Establece La Cartera De Servicios Comunes Del Sistema Nacional de Salud y El Procedimient. Madrid: Boletín Of Del Estado (2014).

[39] Martín-López R Hernández-Barrera V De Andres AL Carrasco-Garrido P De Miguel AG Jimenez-Garcia R. Trend in cervical cancer screening in Spain (2003–2009) and predictors of adherence. Eur J Cancer Prev. (2012) 21:82–8. 10.1097/CEJ.0B013E32834A7E46 22129658

[40] Bosetti C Bertuccio P Malvezzi M Levi F Chatenoud L Negri E. Cancer mortality in Europe, 2005–2009, and an overview of trends since 1980. Ann Oncol Off J Eur Soc Med Oncol. (2013) 24:2657–71. 10.1093/ANNONC/MDT301 23921790

[41] Cohen PA Jhingran A Oaknin A Denny L. Cervical cancer. Lancet. (2019) 393:169–82. 10.1016/S0140-6736(18)32470-X 30638582

[42] Jansen EEL Zielonke N Gini A Anttila A Segnan N Vokó Z. Effect of organised cervical cancer screening on cervical cancer mortality in Europe: a systematic review. Eur J Cancer. (2020) 127:207–23. 10.1016/J.EJCA.2019.12.013 31980322

[43] Liontos M Kyriazoglou A Dimitriadis I Dimopoulos MA Bamias A. Systemic therapy in cervical cancer: 30 years in review. Crit Rev Oncol Hematol. (2019) 137:9–17. 10.1016/J.CRITREVONC.2019.02.009 31014518

[44] Grau JF Farinas-Madrid L Garcia-Duran C Garcia-Illescas D Oaknin A. Advances in immunotherapy in cervical cancer. Int J Gynecol Cancer. (2023) 33:403–13. 10.1136/IJGC-2022-003758 36878562

[45] Haelens A Roche L Bastos J Woronoff AS Zorzi M Francart J. Trends in net survival from cervical cancer in six European Latin countries: results from the SUDCAN population-based study. Eur J Cancer Prev. (2017) 26:S92–9. 10.1097/CEJ.0000000000000292 28005611

[46] Svahn MF Munk C Von Buchwald C Frederiksen K Kjaer SK. Burden and incidence of human papillomavirus-associated cancers and precancerous lesions in Denmark. Scand J Public Health. (2016) 44:551–9. 10.1177/1403494816653669 27289104

[47] Shack L Lau HY Huang L Doll C Hao D. Trends in the incidence of human papillomavirus–related noncervical and cervical cancers in Alberta, Canada: a population-based study. C Open. (2014) 2:E127. 10.9778/CMAJO.20140005 25114894 PMC4117359

[48] Choi I Lee D Son KB Bae S. Incidence, cost and gender differences of oropharyngeal and noncervical anogenital cancers in South Korea. BMC Public Health. (2020) 20:1–11. 10.1186/S12889-020-09161-Y/TABLES/5 32600300 PMC7325282

[49] Buttmann-Schweiger N Klug SJ Luyten A Holleczek B Heitz F Du Bois A. Incidence patterns and temporal trends of invasive nonmelanotic vulvar tumors in Germany 1999–2011. A population-based cancer registry analysis. PLoS ONE. (2015) 10:8073. 10.1371/JOURNAL.PONE.0128073 26020540 PMC4447423

[50] Tanaka Y Ueda Y Kakuda M Yagi A Okazawa A Egawa-Takata T. Trends in incidence and long-term survival of Japanese women with vulvar cancer: a population-based analysis. Int J Clin Oncol. (2019) 24:1137–42. 10.1007/S10147-019-01453-7 31025128

[51] Khadraoui H Thappa S Smith M Davidov A Castellanos MR. Age-associated trends of vulvar cancer in the US. Menopause. (2020) 28:119–25. 10.1097/GME.0000000000001687 33109996

[52] Bray F Laversanne M Weiderpass E Arbyn M. Geographic and temporal variations in the incidence of vulvar and vaginal cancers. Int J Cancer. (2020) 147:2764–71. 10.1002/IJC.33055 32410226

[53] Holleczek B Sehouli J Barinoff J. Vulvar cancer in Germany: increase in incidence and change in tumour biological characteristics from 1974 to 2013. Acta Oncol. (2018) 57:324–30. 10.1080/0284186X.2017.1360513 28799431

[54] Razzaghi H Saraiya M Thompson TD Henley SJ Viens L Wilson R. Five-year relative survival for human papillomavirus-associated cancer sites. Cancer. (2018) 124:203–11. 10.1002/CNCR.30947 29105738 PMC5793215

[55] Senkomago V Henley SJ Thomas CC Mix JM Markowitz LE Saraiya M. Human Papillomavirus-Attributable Cancers - United States, 2012–2016. MMWR Morb Mortal Wkly Rep. (2019) 68:724–8. 10.15585/MMWR.MM6833A3 31437140 PMC6705893

[56] Hansen BT Campbell S Nygård M. Long-term incidence trends of HPV-related cancers, and cases preventable by HPV vaccination: a registry-based study in Norway. BMJ Open. (2018) 8:e019005. 10.1136/BMJOPEN-2017-019005 29476028 PMC5855252

[57] Deshmukh AA Suk R Shiels MS Sonawane K Nyitray AG Liu Y. Recent Trends in Squamous Cell Carcinoma of the Anus Incidence and Mortality in the United States, 2001-2015. J Natl Cancer Inst. (2020) 112:829–38. 10.1093/JNCI/DJZ219 31742639 PMC7825484

[58] Ferlay J Colombet M Soerjomataram I Mathers C Parkin DM Piñeros M. Estimating the global cancer incidence and mortality in 2018: GLOBOCAN sources and methods. Int J Cancer. (2019) 144:1941–53. 10.1002/IJC.31937 30350310

[59] Guren MG Aagnes B Nygård M Dahl O Møller B. Rising incidence and improved survival of anal squamous cell Carcinoma in Norway, 1987–2016. Clin Colorectal Cancer. (2019) 18:e96–103. 10.1016/J.CLCC.2018.10.001 30415990

[60] Daubisse-Marliac L Colonna M Trétarre B Defossez G Molinié F Jéhannin-Ligier K. Long-term trends in incidence and survival of penile cancer in France. Cancer Epidemiol. (2017) 50:125–31. 10.1016/J.CANEP.2017.08.014 28898817

[61] Hansen BT Orumaa M Lie AK Brennhovd B Nygård M. Trends in incidence, mortality and survival of penile squamous cell carcinoma in Norway 1956-2015. Int J Cancer. (2018) 142:1586–93. 10.1002/IJC.31194 29205336 PMC5838782

[62] Trama A Foschi R Larrañaga N Sant M Fuentes-Raspall R Serraino D. Survival of male genital cancers (prostate, testis and penis) in Europe 1999-2007: Results from the EUROCARE-5 study. Eur J Cancer. (2015) 51:2206–16. 10.1016/J.EJCA.2015.07.027 26421823

[63] World Health Organization. Sexually Transmitted Infections (STIs). Fact Sheets. (2022). Available online at: https://www.who.int/news-room/fact-sheets/detail/sexually-transmitted-infections-(stis) (accessed January 9, 2023).

[64] Kombe Kombe AJ Li B Zahid A Mengist HM Bounda GA Zhou Y. Epidemiology and burden of human papillomavirus and related diseases, molecular pathogenesis, and vaccine evaluation. Front Public Heal. (2021) 8:28. 10.3389/FPUBH.2020.552028 33553082 PMC7855977

[65] Krzowska-Firych J Lucas G Lucas C Lucas N Pietrzyk Ł. An overview of Human Papillomavirus (HPV) as an etiological factor of the anal cancer. J Infect Public Health. (2019) 12:1–6. 10.1016/J.JIPH.2018.06.005 29980478

[66] Rosenblum HG Lewis RM Gargano JW Querec TD Unger ER Markowitz LE. Human papillomavirus vaccine impact and effectiveness through 12 years after vaccine introduction in the United States, 2003 to 2018. Ann Intern Med. (2022) 175:918–26. 10.7326/M21-3798 35576590 PMC11614147

[67] Eldridge RC Schiffman M Wentzensen N Pawlita M Waterboer T Wilson L. Smoking and subsequent human papillomavirus infection: a mediation analysis. Ann Epidemiol. (2017) 27:724–30. 10.1016/J.ANNEPIDEM.2017.10.004 29107447 PMC5705255

[68] Asiaf A Ahmad ST Mohammad SO Zargar MA. Review of the current knowledge on the epidemiology, pathogenesis, and prevention of human papillomavirus infection. Eur J Cancer Prev. (2014) 23:206–24. 10.1097/CEJ.0B013E328364F273 24129107

[69] Bruni L Saura-Lázaro A Montoliu A Brotons M Alemany L Diallo MS. HPV vaccination introduction worldwide and WHO and UNICEF estimates of national HPV immunization coverage 2010–2019. Prev Med. (2021) 144:106399. 10.1016/J.YPMED.2020.106399 33388322

[70] Reitsma MB Kendrick PJ Ababneh E Abbafati C Abbasi-Kangevari M Abdoli A. Spatial, temporal, and demographic patterns in prevalence of smoking tobacco use and attributable disease burden in 204 countries and territories, 1990–2019: a systematic analysis from the Global Burden of Disease Study 2019. Lancet. (2021) 397:2337–60. 10.1016/S0140-6736(21)01169-7 34051883 PMC8223261

[71] UNESCO Institute for Statistics (UIS). SDG 4 Data Book: Global Education Indicators 2019. Montreal, QC: UNESCO-UIS (2019).

[72] Allen CT Lewis JS El-Mofty SK Haughey BH Nussenbaum B. Human papillomavirus and oropharynx cancer: biology, detection and clinical implications. Laryngoscope. (2010) 120:1756–72. 10.1002/LARY.20936 20669304

