# Supplementary material for: Epidemiology of human papillomavirus-associated anogenital cancers in Granada: a three-decade population-based study
Source: Front Public Health. 2023 Sep 14;11:1205170. doi: 10.3389/fpubh.2023.1205170 (PMC10537955; doi:10.3389/fpubh.2023.1205170)

**TABLE S1:** Sex-stratified 1, 3 and 5-year survival rates for anal cancer in the province of Granada during the period 2008-2017. *OS: overall survival; NS: Net survival; ASNS: age-standardized net survival. Probabilities in percent (%).

|  |  |  | | | | | | | | | | | |
| --- | --- | --- | --- | --- | --- | --- | --- | --- | --- | --- | --- | --- | --- |
|  |  | **2008-2017** | | | | | | | | | | | |
|  |  |  | ***1-year*** | | |  | ***3-years*** | | |  | ***5-years*** | | |
|  | **Cases at risk** |  | **OS** | **NS (95%CI)** | **ASNS (95%CI)** |  | **OS** | **NS (95%CI)** | **ASNS (95%CI)** |  | **OS** | **NS (95%CI)** | **ASNS (95%CI)** |
| **Men** | 39 |  | 72 | 74 (56-86) | 75 (58-86) |  | 44 | 47 (29-63) | 51 (39-63) |  | 35 | 40 (22-57) | 45 (32-58) |
| **Women** | 34 |  | 62 | 62 (43-77) | 64 (50-75) |  | 50 | 52 (33-67) | 54 (38-68) |  | 42 | 45 (26-62) | 47 (29-63) |

**FIGURE S1:** Projected age-standardized incidence rates (ASIR-E) of anogenital cancers in the province of Granada for the period 2018-2022.


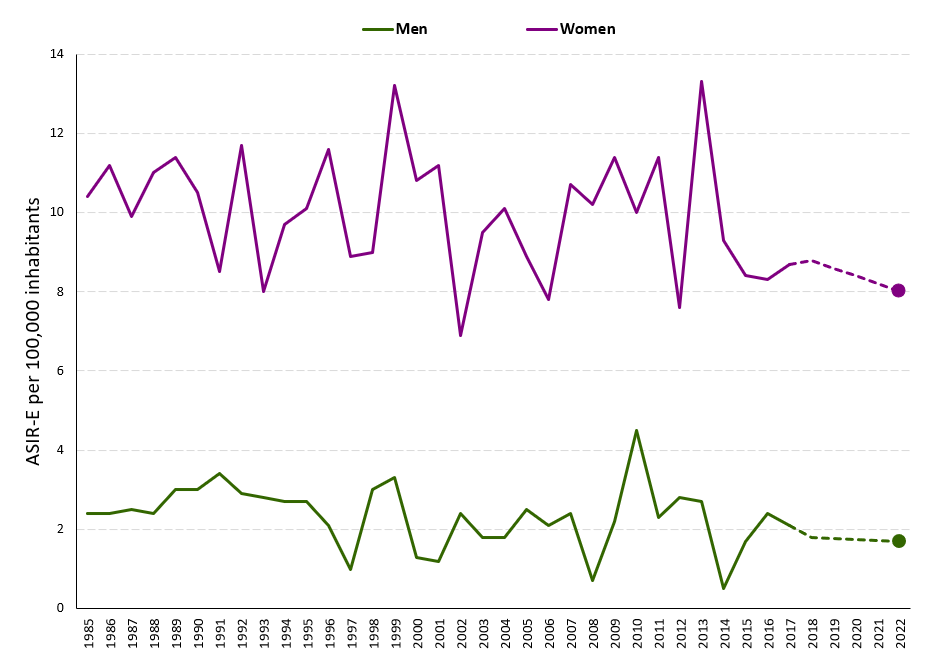


**FIGURE S2:** Spatial distribution of age-standardized incidence rates (ASIR-E) of anogenital cancers in the province of Granada for the period 2008-2017, by health district.


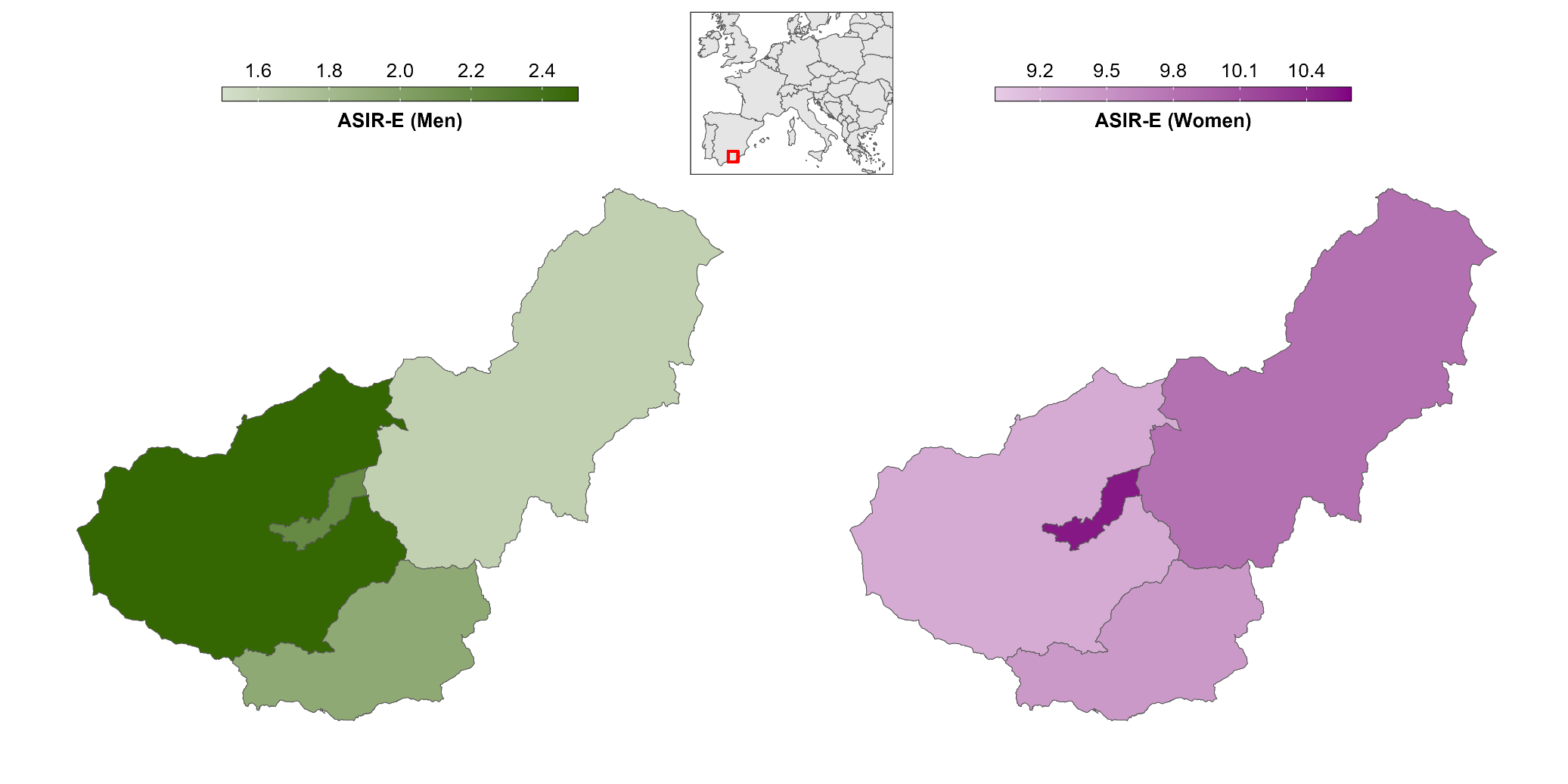

Supplement: Supplementary file 1 [file Data_Sheet_1.docx]
